# Supplementary material for: Comparative Efficacy of Parenteral and Mucosal Recombinant Probiotic Vaccines Against SARS-CoV-2 and S. pneumoniae Infections in Animal Models
Source: Vaccines (Basel). 2024 Oct 19;12(10):1195. doi: 10.3390/vaccines12101195 (PMC11512341; doi:10.3390/vaccines12101195)
Supplement: Supplementary file 1 [file vaccines-12-01195-s001.zip › vaccines-3217564-supplementary.pdf]

Supplementary

Table S1. - Evaluation of the level of virus-neutralizing activity in Syrian golden hamsters by inhibition of protein S binding to human ACE 2 in ELISA.

Experimental groups	Sample number*	The neutralization index of serum **		The neutralization index of swabs**	
L3-S1	1	25.2	22.4±7.7	14.5	13.1±3.9
	2	37.5		19.7	
	3	23.8		14.8	
	4	4.8		6.8	
	5	20.7		9.6	
L3	11	17.5	14.1±6.2	21.4	17.5±5.2
	12	11.9		14.5	
	13	26.1		26.7	
	14	7.9		16.2	
	15	7.1		8.7	
S1	11	10,1	13,7±1,6	14,8	5,4±3,8
	12	17,7		4,3	
	13	13,3		0,5	
	14	13,7		4,7	
	15	13,5		2,5	
Untreated control	21	1.8	5.9±2.1	5.5	5.3±1.7
	22	7.9		5.6	
	23	7.1		2.0	
	25	7.1		8.2	
